# Multiscale Imaging of Human Adipose Tissue: A Neglected Partner in Proteinuria Linked to Obesity

**DOI:** 10.3390/biomedicines13112719

**Published:** 2025-11-06

**Authors:** Davide Viggiano, Erica Bortone, Salvatore Tolone, Francesco Saverio Lucido, Claudio Gambardella, Giusiana Nesta, Giuseppe Gigliotti, Michelangelo Nigro, Maddalena Paolicelli, Vittorio D’Orlando, Ludovico Docimo

**Affiliations:** 1 Division of General, Oncological, Mini-Invasive and Obesity Surgery, University of Study of Campania “Luigi Vanvitelli”, via Luigi Pansini n° 5, 80131 Naples, Italy; erica.bortone@studenti.unicampania.it (E.B.); salvatore.tolone@unicampania.it (S.T.); francescosaverio.lucido@unicampania.it (F.S.L.); claudio.gambardella2@unicampania.it (C.G.); giusiana.nesta@studenti.unicampania.it (G.N.); maddalena.paolicelli@studenti.unicampania.it (M.P.);; 2 UOC Nephrology, Eboli Hospital, 84025 Eboli, Italy; g.gigliotti@aslsalerno.it (G.G.); michelangelonigro@hotmail.it (M.N.)

**Keywords:** adipocytes, AFM, nephrotic syndrome

## Abstract

Nephrotic syndrome (NS) is a systemic disorder characterized not only by glomerular dysfunction but also by profound dysregulation of lipid metabolism and microvascular integrity. Adipose tissue, as a central lipid-handling and endocrine organ, undergoes structural and functional remodeling in chronic renal conditions yet remains underexplored in this context. The aim of this manuscript is to integrate adipose tissue imaging into the diagnostic and mechanistic framework of NS. To establish this perspective, we first summarize current knowledge on adipose tissue architecture and imaging in both physiological states and renal disease. We then present a multimodal imaging approach—combining ultrasound (US), histology, and atomic force microscopy (AFM)—applied to human adipose tissue as a potential diagnostic and pathophysiological marker in NS. Original imaging from our laboratory experience is presented as a demonstrative material, complemented by literature synthesis. Given that different modalities of imaging-based characterization of adipose tissue are sparse across the literature, this pictorial review offers a guide to identifying structural biomarkers of adipose remodeling in NS. By bridging imaging modalities with metabolic and vascular perturbations observed in NS, this work aims to guide future research toward the clinical application of adipose tissue imaging in renal disease. This provides insights into cell size heterogeneity, vascular topology, and subcellular features such as membrane wrinkles and nanodomain organization. We propose that such morphometric parameters, accessible via minimally invasive biopsies, could serve as surrogate markers of adipose remodeling in nephrotic syndrome. This sets the stage for integrating adipose tissue imaging into the diagnostic and mechanistic evaluation of systemic features in NS.

## 1. Introduction

Nephrotic syndrome (NS) affects 3 in 100,000 adults per year [1] and has a prevalence of 16 in 100,000 children [2]. It is defined by proteinuria > 3.5 g/day, hypoalbuminemia, edema, a prothrombotic state, and hyperlipidemia, with podocyte injury at its core.

Nephrotic syndrome is increasingly recognized as a systemic condition that affects peripheral tissues beyond the kidney, including adipose tissue. Human studies in chronic kidney disease demonstrate that adipose tissue undergoes inflammatory activation, characterized by increased macrophage infiltration and reduced adipocyte size, indicative of heightened lipolytic and catabolic activity [3]. These alterations mirror the systemic hypercatabolic state seen in nephrotic syndrome [4] and suggest that adipose tissue plays an active role in mediating metabolic dysfunction. Experimental models confirm that uremic and proteinuria conditions are associated with adipose tissue remodeling. Indeed, in animal models of kidney disease, including the 5/6 nephrectomy model, dynamic contrast-enhanced magnetic resonance imaging (MRI) reveals hypoperfusion of adipose depots accompanied by adipocyte degeneration [5].

Among the metabolic derangements in NS is hyperuricemia, which affects 37–45% of NS patients [6,7]. Elevated serum uric acid has been associated with increased visceral adiposity [8], possibly through direct secretion from adipocytes [9], and consequent proinflammatory milieu [10]. However, urate-lowering therapy in asymptomatic hyperuricemia has a small effect on glomerular protection, difficult to reproduce among studies [11,12,13,14,15]. However, the results regarding the effects of urate-lowering drugs (allopurinol and febuxostat) on proteinuria are conflicting among studies [16,17,18]. In Adriamycin-treated rodents, a model of nephrotic syndrome, allopurinol reduced proteinuria [19,20].

In Adriamycin-induced glomerular injury, adipose-derived stromal cells have been shown to ameliorate nephropathy by promoting neovascularization and reducing fibrosis [21], directly linking adipose tissue biology to kidney repair capacity. Adriamycin treatment induces glucose intolerance and loss of skeletal muscle mass [22], possibly mediated by PED/PEA-15 in lipidic tumors [23,24,25]. Adriamycin also activates calcium/calmodulin-dependent protein kinase II (CaMKII) [26] a calcium-binding protein expressed in the heart, kidney, brain [27], and adipose tissue [28]. This kinase mediates downstream effects of TGF-β1 and calcium flux, both of which are elevated in nephrotic syndrome, suggesting that CaMKII may represent a shared molecular driver of adipose remodeling and glomerular dysfunction. MRI–proton density fat fraction measurements further demonstrate that renal and hepatic fat correlates with albuminuria and kidney outcomes in metabolic disease [29], supporting the use of imaging biomarkers to detect early pathological fat remodeling before overt renal decline.

Taken together, these observations suggest clear translational pathways for adipose imaging in nephrotic syndrome. Ultrasound-based mesoscopic texture analysis may provide a non-invasive surrogate for adipocyte organization and local stiffness, capturing early metabolic changes. Histology-based capillary topology and AFM-derived surface biomechanics offer sensitive readouts of endothelial dysfunction and ECM remodeling, which are central to nephrotic syndrome pathogenesis in both humans and Adriamycin models. Furthermore, these metrics could be used longitudinally to monitor treatment response to corticosteroids, renin–angiotensin–aldosterone blockade, or experimental antivascular therapies. As fibrosis and microvascular injury are major determinants of prognosis, combining depot-specific imaging biomarkers with organ-level MRI fat metrics may improve patient stratification, enable early diagnosis, and guide precision therapy in nephrotic syndrome.

As further discussed below, magnetic resonance imaging (MRI) and computed tomography (CT) are established tools for assessing adipose tissue distribution and volume in both clinical and experimental research. CT provides high-resolution, quantitative estimates of total and regional fat depots by distinguishing adipose tissue based on Hounsfield units, enabling accurate measurement of visceral, subcutaneous, and perirenal fat compartments. This technique has been widely used to quantify renal sinus fat and to explore its association with renal hemodynamics and chronic kidney disease risk [30]. MRI and techniques such as proton density fat fraction (PDFF), Dixon imaging, relaxometry, and MR spectroscopy allow for precise measurement of lipid and water contents. These methods allow us to quantify kidney fat accumulation in metabolic syndrome or CKD, as well as distinguishing cortical and medullary lipid accumulation [29,31]. Large population-based MRI analyses showed a link between higher kidney volume and reduced visceral adiposity [32].

Furthermore, dynamic contrast-enhanced and T2*-weighted MRI in a CKD model revealed hypoperfusion and adipocyte degeneration in the infrapatellar fat pad [5]. Finally, MRI-derived data highlighted adipose infiltration as a contributor to sarcopenia [33]. These imaging modalities can be complemented by higher-resolution approaches such as high-frequency ultrasound, histology, and atomic force microscopy.

### Background

In NS, the loss of proteins with urine causes a reduction in plasma albumin. The syndrome does not necessarily occur at the threshold of proteinuria of 3.5 g/day, being dependent on the liver synthesis rate of plasma proteins and likely on other unknown factors [34].

If S_alb_ is the liver synthesis rate of albumin (12–14 g/die at basal lever), Cl_alb_ is the metabolic clearance of albumin (L/die), U_alb_ is the urinary loss of albumin (g/die), and [Alb] is the plasma concentration of albumin (g/L), the following summarizes the condition of nephrotic syndrome [34]:

d[Alb]/dt ∗ V_p_ = S_alb_ − Cl_alb_ ∗ [Alb] − U_alb_ (1)

where V_p_ is the plasma volume.

At the steady state (d[Alb]/dt = 0), the following can be derived:

[Alb] = (S_alb_ − U_alb_)/Cl_alb_ (2)

More complex models are available (used for peritoneal dialysis, e.g., [35]) that also consider the extravascular albumin, though these fall outside of the scope of the review.

Overall, Equation (1) explains that in proteinuria and nephrotic syndrome (increased U_alb_), the liver must react with increased synthesis of albumin (S_alb_):

S_alb_ = [Alb] ∗ Cl_alb_ + U_alb_

However, the liver is not specific to this reaction and also increases the synthesis of low-density lipoproteins (LDLs) [36], leading to dyslipidemia, hypercholesterolemia, and, finally, lipiduria.

This can be synthesized by the equation below:

d[LDL]/dt ∗ V_p_ = α ∗ (S_alb_) − Cl_LDL_ ∗ [LDL]

That is, the LDL concentration increases as a function of albumin synthesis and decreases over time due to metabolic clearance (Cl_LDL_) [37].

This is notable because NS is also associated with carotid intima-media thickening, even in children [38]. The latter observation lends support to the hypothesis that LDLs are mediators of accelerated aging on arterial walls [39]. It is surprising that we lack any evidence of the usefulness of statins to revert or slow down the arterial aging in nephrotic syndrome [39].

In nephrotic syndrome, the plasma lipoprotein increase is of the atherogenic type (LDLs and very-low-density lipoproteins, VLDLs) [40], whereas lipiduria is enriched in small high-density lipoproteins (HDLs) [41], possibly because of their smaller size, which means that they can more easily pass through the glomerular filter.

We will now explore the parallel between nephrotic syndrome and the widely known dyslipidemia linked to obesity (Table 1).

Nephrotic syndrome and obesity share the same dyslipidemic profile, increased risk of chronic kidney disease (CKD), and an altered urinary lipid profile.

In severe obesity, the renal hemodynamic response to a protein load is delayed, indicating impaired adaptive vasodilation and altered intraglomerular pressure regulation [44]. As nephrotic syndrome shares key metabolic features with obesity, including dyslipidemia, endothelial injury, and adipokine imbalance, it is plausible that similar adipose–renal cross-talk contributes to renal microvascular dysregulation in nephrotic states.

An underrecognized, peculiar form of nephrotic syndrome is that associated with obesity [45,46]. In single case reports, the use of bariatric surgery was able to reverse nephrotic syndrome [47]. Obesity is associated with proteinuria [45] and reduced glomerular filtration [44]. Due to the increasing prevalence of obesity in the population, the medical community is now devoting greater attention to obesity-related glomerular damage [48].

The link between obesity and proteinuria is of particular interest because NS triggers a cascade of systemic changes, including alterations in lipid metabolism, inflammatory responses, and vascular function [36]. These systemic manifestations implicate peripheral tissues—including adipose tissues—as both targets and modulators of disease processes [36].

In other words, NS is a peculiar model of dyslipidemia accompanying obesity without the overall increase in body fat.

**Figure 1:**
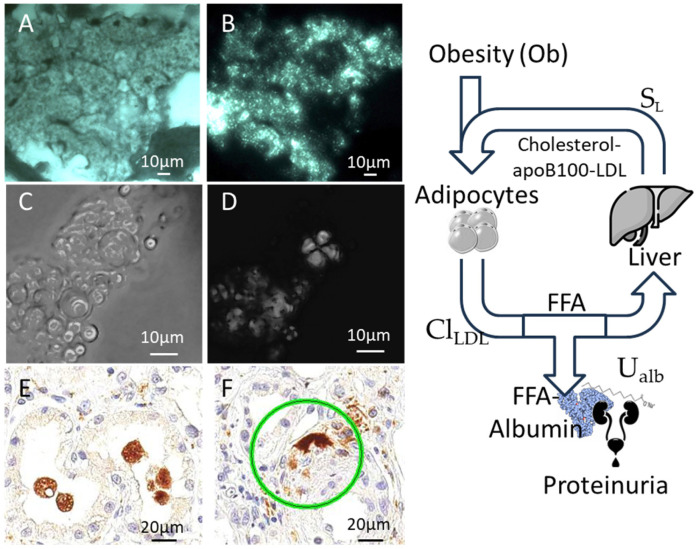
Foamy cells in nephrotic syndrome. (**A**,**B**) Tubule cells from the cryostat section of a kidney biopsy in a patient with nephrotic syndrome. The cytoplasm is filled by vacuoles (**A**), which show typical birefringence under polarized light (**B**) showing that the vacuolar degeneration is due to the accumulation of lipidic droplets. (**C**,**D**) A urine cast from the same patient showing the presence of numerous vesicles (**C**), which demonstrate birefringence under polarized light (**D**). (**E**,**F**) The vacuolated cells detach and fall in the tubular lumen and are positive for CD68 in immunohistochemistry, a marker of macrophages (the green ring indicates an intertubular CD68 positive element). The panel on the right summarizes the relationship between proteinuria, obesity, and dyslipidemia (see text for equations). Cl_LDL_: clearance of LDL; S_L_: liver synthesis of lipoproteins; U_Alb_: urinary loss of albumin; FFA-albumin: free fatty acids transported by albumin.

Several histopathologic alterations can lead to proteinuria in the nephrotic range, such as diabetic nephropathy, minimal change disease (MCD, the most frequent form in childhood), membranous glomerulonephritis (MG), focal segmental glomerulosclerosis (FSGS), and amyloid disease. In adulthood, the pathogenesis is immunological, with evidence of anti-phospholipase A2-receptor (PLA2R) in MN [49] and circulating anti-nephrin antibodies in MCD and FSGS [50]. At variance, in children with steroid-resistant nephrotic syndrome, the pathogenesis is genetic [51]. An almost universal hallmark of high proteinuria, irrespective of the cause, is the presence of “foamy cells”, which represent the vacuolar degeneration of tubular cells (Figure 1). Although the origin of these cells is unclear, the vacuoles in foamy cells are filled with lipids, as evidenced by polarized light observation (Figure 1), and are positive for the macrophage marker CD-68. This represents another similarity with obesity: foamy cells, in fact, are also present in atherosclerotic plaques [52].

It is relevant here to note that the adipose tissue mass is not increased in NS, and there is even a non-significant trend towards a its reduction compared to the non-nephrotic state [4].

Therefore, on one hand, the common dyslipidemic profile in NS and obesity is linked to similar histological findings (foamy cells, kidney damage). On the other hand, dyslipidemia is due to a change in liver function driven by hypertrophic adipose tissue in obesity and by hypoalbuminemia in NS. Because LDL cholesterol is due to a balance between its storage in adipocytes and its synthesis in the liver (Figure 1), adipose tissue undergoes functional changes in nephrotic syndrome.

This cycle can also be represented as follows:

d[LDL]/dt = S_L_ ([FFA], U_alb_) − Cl_LDL_ (Ob) ∗ [LDL]/V_p_ (3)

where [LDL] is the plasma concentration of LDL cholesterol. This increases with the hepatic synthesis of LDL (S_L_), which in turn is a function of the concentration of free fatty acids ([FFA]) and albumin loss (U_alb_). LDLs decrease with their clearance by adipocytes (Cl_LDL_), which depends on their total amount in obesity (Ob). The loss of albumin in NS increases S_L_.

The dynamic relationships described in Equations (1)–(3), involving feedback between albumin loss, hepatic lipoprotein synthesis, and adipocyte lipid handling, represent a nonlinear system that may exhibit sensitivity to initial conditions, a hallmark of chaotic dynamics. This inherent instability of chaotic systems supports the application of multiscale imaging approaches and analyses such as Lyapunov exponents [53], which allow the direct visualization of emergent structural patterns that cannot be inferred from steady-state biochemical measures alone.

As is evident in Figure 1 and Equation (3), adipocytes play a central role in obesity and NS.

Despite its central role in lipid homeostasis and endocrine signaling, the structural and microvascular alterations in adipose tissue in NS remain poorly characterized.

Adipose tissue plays a central role in energy storage, metabolic regulation, and endocrine signaling, with profound implications for obesity, diabetes, and cardiovascular disease [54]. It is highly plastic, and its hypertrophy and structure can be rearranged after bariatric surgery [55]. Despite its physiological importance, the intricate architecture of adipose tissue—spanning cellular organization, microvascular networks, and extracellular matrix components—remains incompletely characterized across multiple spatial scales. This knowledge gap limits our understanding of adipose tissue remodeling in health and disease.

Traditional histological approaches provide valuable cellular-level insights but are often limited by tissue sectioning artifacts and insufficient spatial context. Recent technological advances now allow for multiscale imaging of adipose tissue structure with unprecedented resolution. Ultrasound (US), histological methods, and atomic force microscopy (AFM) can reveal architectural features ranging from whole-tissue organization to nanoscale surface characteristics. These imaging modalities provide valuable morphometric and biomechanical insights that may reflect the systemic state of lipid and vascular regulation, both disrupted in NS.

In this review, we synthesize the current understanding of adipose tissue structure as seen with US, Histology, and AFM. We then present original imaging data using these modalities, showcasing their potential to detect subtle structural changes relevant to nephrotic syndrome. Our multimodal approach ranges from mesoscopic tissue heterogeneity to submicron structural domains, elucidating adipocyte morphology, capillary networks, and extracellular matrix organization with unprecedented detail.

This atlas provides novel insights into adipose tissue microanatomy, highlights technical advances in label-free imaging, and establishes a versatile platform for investigating adipose remodeling in metabolic disorders. By correlating complementary imaging modalities, we reveal new structural biomarkers potentially relevant to clinical diagnostics and fundamental biology.

Our goal is to frame adipose imaging as a novel tool for studying and potentially monitoring systemic manifestations of renal disease.

## 2. Adipose Tissue Structure and Imaging Modalities

At present, several tools are available to characterize the adipose structure in vivo. Table 2 summarizes these tools. While widely used tools such as dual-energy X-ray absorptiometry (DXA), bioimpedentiometry, CT scan, MRI, and standard ultrasound can provide an estimate of the total adipose mass in the human body, their usefulness in interrogating changes in adipocyte function remains limited.

Classical histological methods require adipose tissue and are therefore invasive. They provide information regarding the size of single adipose cells, which is an advancement compared to the total body fat.

However, the advent of new technologies such as artificial intelligence image analysis, ultrasound speckle tracking, and atomic force microscopy, coupled with metabolomic tools, will certainly revolutionize our understanding of adipose tissue in NS.

### 2.1. Ultrasound Imaging of Adipose Tissue

Ultrasound is a widely accessible, non-invasive modality that can reveal the mesoscopic architecture of subcutaneous and visceral fat. Adipose tissue typically appears hypoechoic with scattered echogenic speckles corresponding to connective tissue septa or microvascular structures. High-frequency probes (e.g., linear 4–15 MHz) enhance the resolution and can detect variability in cell size and packing density, particularly useful for monitoring obesity and lipodystrophy states. Emerging evidence suggests that adipose tissue echotexture may reflect metabolic health, inflammation, and vascularization, all relevant to NS pathophysiology.

### 2.2. Histological Analysis of Adipose Tissue

Histology remains the gold standard for assessing adipocyte morphology, size distribution, and microvascular context. Classic staining techniques (e.g., Hematoxylin and Eosin, Masson’s trichrome) are supplemented by immunohistochemistry for endothelial or fibrotic markers. Innovations in tissue processing, including thick cryosectioning and defatting protocols, have enabled better preservation of adipocyte shape and capillary details. Polarized light enhances the contrast of vascular structures, particularly when red blood cells are absent. Quantitative tessellation analyses (e.g., Voronoi diagrams) allow the objective evaluation of cell packing and topology.

### 2.3. Atomic Force Microscopy (AFM)

AFM offers nanometer-resolution surface topography and mechanical data on adipocyte membranes and the extracellular matrix. Despite challenges in imaging lipid-rich tissues, cryopreservation followed by chemical defatting facilitates AFM probing. Surface features such as wrinkles, granularity, and nanodomains may reflect lipid compartmentalization, membrane tension, or structural remodeling. AFM thus complements histology and US by revealing the fine architecture of adipocytes and their microenvironment.

## 3. A Multiscale Imaging Atlas to Guide Adipose Tissue Research in Nephrotic Syndrome

### 3.1. Ultrasound Reveals Mesoscopic Heterogeneity in Post-Operative Samples

Human adipose tissue samples can be obtained from surgical procedures or kidney biopsies and imaged by ultrasound, histology (whole-slide imaging with AI), and atomic force microscopy. The images can be compared and completed with in vivo imaging of subcutaneous fat by ultrasound. Following standard fixation and cryoprotection, samples can be imaged using high-frequency B-mode ultrasound (≈12–15 MHz) to visualize mesoscopic adipose architecture both in vivo and ex vivo. High-frequency ultrasound enables clear visualization of adipose depots, septa, and adjacent muscle layers, revealing characteristic granular echotexture corresponding to adipocyte organization. Image processing with open-source software (ImageJ version 1.53) and Voronoi-based analysis was used to infer cell boundaries and spatial heterogeneity from the speckle pattern, providing structural information without invasive sectioning.

**Figure 2:**
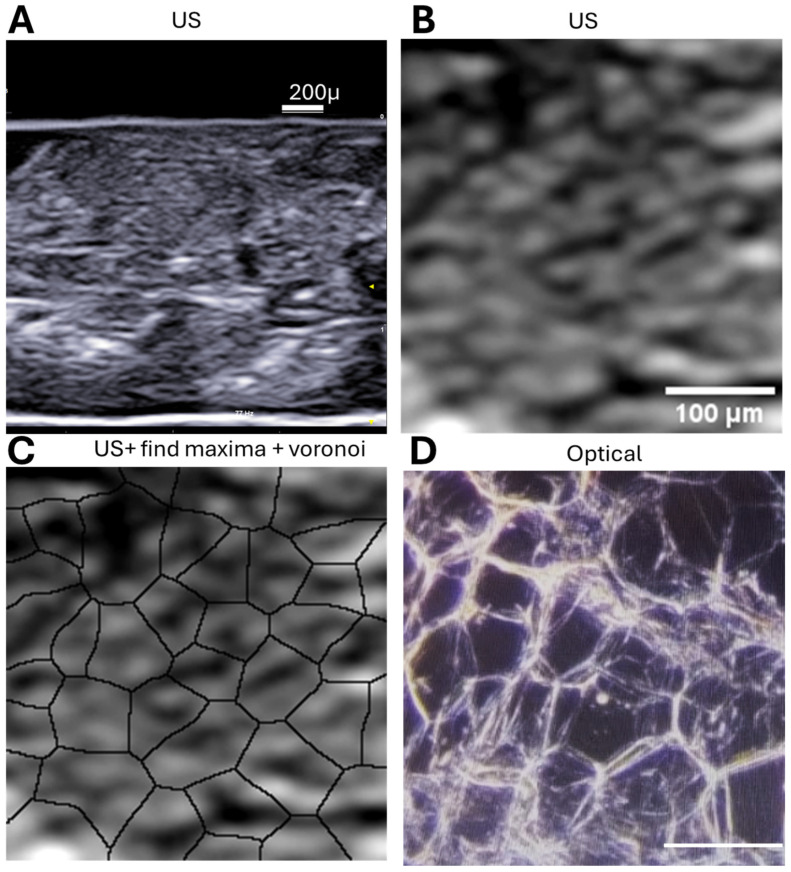
Multiscale visualization of adipose tissue using ultrasound and histology. (**A**) B-mode ultrasound image of a human subcutaneous adipose tissue sample acquired with a 15 MHz linear probe (Esaote L4–15). The triangle indicates the position of US beam focus; (**B**) Enlarged view of the boxed region in (**A**), highlighting the echogenic speckle pattern characteristic of adipocyte borders. (**C**) Application of the Voronoi algorithm in ImageJ to (**B**), revealing polygonal segmentation that corresponds to individual adipocytes (same magnification as **B**). (**D**) Cryosection of the same tissue sample stained and imaged at 5× magnification, confirming the presence of similarly sized and shaped adipocytes to those predicted via ultrasound-derived Voronoi segmentation (same magnification as (**B**); scalebar: 100 micron).

In Figure 2 we compare the processed ultrasound images to histological cryosections from the same sample. The patterns revealed by Voronoi segmentation closely match the morphology of adipocytes observed under low-power light microscopy.

These results support the utility of high-resolution ultrasound, combined with computational image processing, to infer cellular organization in situ without the need for invasive sectioning.

Figure 2A shows a representative US image of subcutaneous adipose tissue, with Figure 2B providing a detailed view. Using ImageJ tools such as “Find Maxima” and Voronoi segmentation (Figure 1C), echogenic speckles are shown to correspond to cell boundaries. Cryosectioned histology of the same sample confirms this (Figure 1D).

### 3.2. Histology Identifies Capillary Networks and Structural Detail

A quick and informative histological analysis of adipose tissue can be obtained through cryoprotection of samples in 10% glucose (after fixation) and then rapid freezing and thick-section imaging of 80 μm thick cryosections obtained using a cryostat at −20 °C. For subsequent imaging, sections need to be subjected to an on-slide defatting protocol involving sequential immersion in xylene, absolute ethanol, acetone, distilled water, and ethanol, followed by air drying. This procedure effectively removes lipid content while preserving tissue morphology. With this method, brightfield and polarized light images can be acquired with a microscope at 5× and 25× objectives. Large-area panoramic stitching can be performed to reconstruct microvascular networks.

This resolves adipocyte morphology, vascular structures, and connective tissue elements. Key observations include the following:

- Adipocyte size and shape heterogeneity between depots;
- ECM fiber alignment and density.

**Figure 3:**
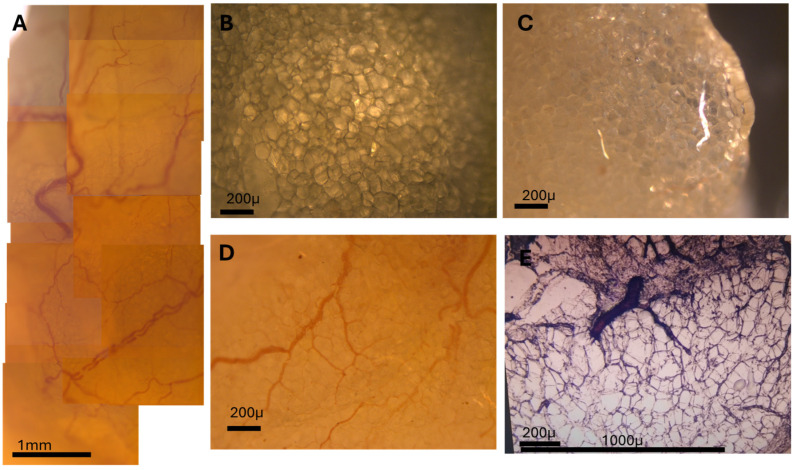
High-resolution visualization of adipose microvasculature using unsectioned and cryosectioned tissue. (**A**) Composite panoramic image showing the microvascularization of adipose tissue across a large area. Capillaries and small arteries are visible traversing the adipocyte field. (**B**) Unsectioned adipose tissue sample imaged under transmitted light before cryosectioning. (**C**) Same field as B, imaged under polarized light. Capillaries devoid of red blood cells show altered birefringence and appear distinctly. (**D**) Detail of a small artery and adjacent capillary network weaving between adipocytes. (**E**) Cryosectioned tissue (80 µm) imaged under brightfield, showing preserved adipocyte structure and microvasculature after on-slide defatting protocol.

By applying cryopreservation, rapid freezing, and on-slide defatting of thick sections (~80 µm), we obtained detailed optical images of intact adipose tissue architecture, shown in Figure 3.

The tissue was cryosectioned using a fast-freezing protocol and then subjected to a sequential solvent treatment (xylene → absolute ethanol → acetone → distilled water → ethanol → air drying) applied directly onto the microscope slide. This technique preserved adipocyte integrity, removed lipid content, and enhanced optical clarity without tissue collapse or tearing.

When imaged under transmitted light and polarized light microscopy, even subtle structures became resolvable. In particular, the microcirculation network—including capillaries and small arteries—was clearly visualized as they traversed between and around adipocytes. Polarized light revealed differential birefringence, particularly highlighting capillaries devoid of red blood cells (RBCs) due to their distinct optical behavior.

By using panoramic image stitching, we reconstructed large composite fields of adipose tissue showing the entire vascular tree, from major vessels to terminal capillaries. This level of detail, achieved without staining or labeling, demonstrates the power of this technique for structural studies of adipose tissue vasculature.

Our cryopreservation and defatting protocol—fast-freezing, then xylene–ethanol–acetone defatting—preserves adipocyte integrity in 80 μm sections. Figure 3A shows a large-area composite of adipose microvascularization. Figure 3B–E highlight polarized light features, capillary paths, and small vessels navigating between adipocytes.

### 3.3. Ultrastructural Features of Fat by AFM

AFM imaging provided nanoscale detail of

- Adipocyte surface texture;
- ECM topography and stiffness variations;
- Differences in mechanical properties across tissue regions or donor types.

Atomic force microscopy (AFM) imaging of cryosectioned and defatted adipose tissue surfaces can reveal three distinct topographical patterns consistently across multiple tissue regions:

1. Flat, grainy regions, characterized by low-amplitude nanoscale texture, interpreted as relatively unstructured interstitial matrix or adipocyte surfaces;
2. Small, irregular wrinkles, possibly corresponding to localized tension lines or collapsed ECM components;
3. Large, elongated wrinkles, running in continuous paths across the tissue surface.

A correlative analysis with optical images acquired at 25× magnification from the same sample regions can be carried out to support the AFM data. The large wrinkles observed in AFM scans correspond precisely to the location of capillary vessels in the optical images. Furthermore, these features displayed periodic constrictions along their length—suggestive of structural striations or flow-induced modulation in the vascular wall.

This correlation confirms that AFM can resolve capillaries at the nanoscale, capturing both their path and wall irregularities. Such structural wrinkles may relate to vessel elasticity, perivascular matrix organization, or post-processing shrinkage effects.

**Figure 4:**
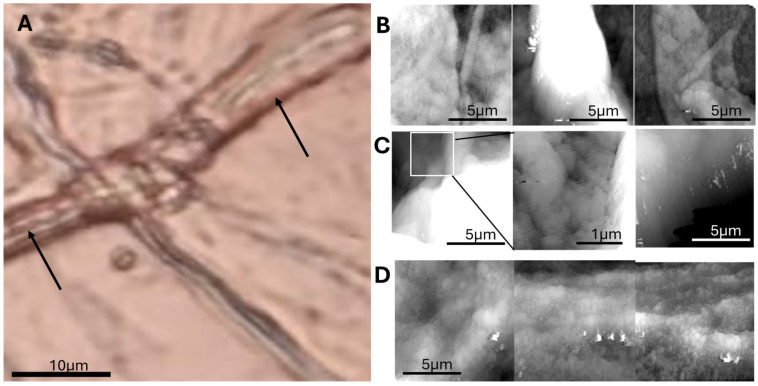
Correlative optical and AFM imaging reveals ultrastructural details of adipose microvasculature. (**A**) Optical image of cryosectioned adipose tissue, showing adipocytes and inter-adipocyte capillaries (arrows). (**B**) AFM topographic image showing grainy surface areas likely corresponding to adipocyte surfaces or extracellular matrix. (**C**) Small-scale wrinkling patterns observed in localized regions, suggestive of microstructural tension. (**D**) Close-up AFM view of a large wrinkle (capillary), showing periodic constrictions along its course, possibly related to vessel wall structure or collapse artifacts.

Three main surface patterns emerged: flat grainy regions, small membrane wrinkles, and large capillary-like folds. Figure 4 compares these features with corresponding optical microscopy.

**Figure 5:**
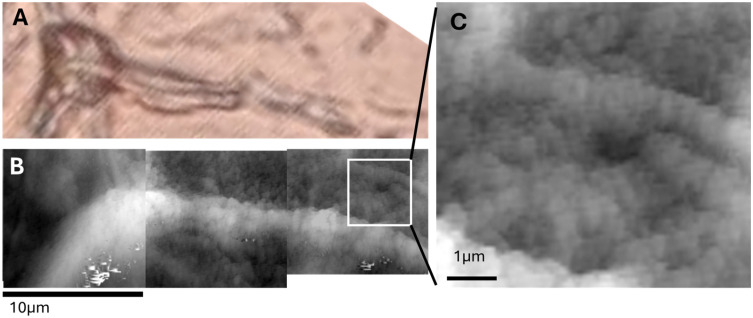
Distinction between membrane artifact wrinkles and capillary structures in adipose tissue. (**A**) Optical image showing both adipocytes and inter-adipocyte capillaries in cryosectioned tissue. (**B**) AFM topography of the same region, showing fine membrane wrinkles over adipocyte surfaces. (**C**) High-magnification AFM view of small, irregular wrinkles likely caused by membrane collapse during defatting. Panels A and B have same magnification and share the same scalebar.

Further analysis of AFM scans reveals that the small wrinkles, initially interpreted as possible ECM tension lines, are more likely membrane collapse artifacts formed during the defatting process. These fine, irregular folds typically appeared over regions corresponding to collapsed adipocytes, particularly where large lipid vacuoles had been removed. This interpretation is supported by the observation that these small wrinkles are confined to the pericellular zones of adipocytes and absent from interstitial regions. Moreover, they lack the structural continuity and regularity of the larger wrinkles associated with capillaries. Figure 5 shows small and large wrinkles.

In regions corresponding to the interior of adipocytes, AFM imaging consistently revealed flat, grainy surfaces characterized by scattered submicron-sized particles. These areas, devoid of large wrinkles, likely represent the collapsed lipid vacuoles after the defatting and dehydration steps. Unlike the smooth lipid globules seen in fresh adipose tissue under optical microscopy, the surfaces imaged by AFM revealed a particulate nanostructure. These granules, typically <1 μm in diameter, may correspond to

1. Residual proteic aggregates (e.g., perilipin remnants or cytosolic protein complexes);
2. Condensed extracellular matrix proteins (e.g., laminin, collagen fragments);
3. Possibly crystallized lipid components resistant to complete solvent extraction.

Optical microscopy confirmed the location of these features as intra-adipocyte, with AFM revealing their fine-scale organization and texture, inaccessible to optical methods.

**Figure 6:**
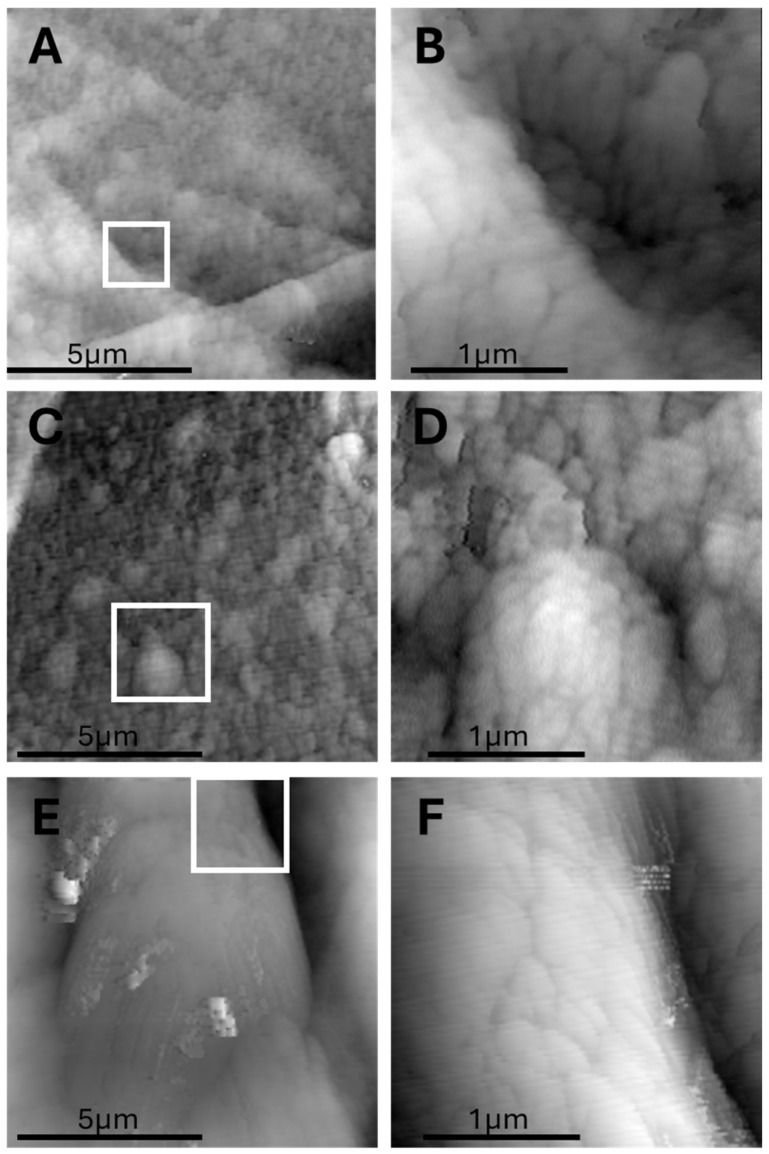
Submicron bulging domains (“AFM domes”) observed on adipocyte surfaces after defatting. (**A**,**C**) AFM topography of an adipocyte interior showing three prominent nanodomes (~200 nm) on a flat background. (**B**,**D**) High magnification highlighting the dome shape and surface continuity. (**E**,**F**) High-magnification scan showing the nanoscopic curvature and well-defined borders of the domes. Each dome appears as a localized, smooth elevation with a low aspect ratio, possibly representing protein–lipid aggregates or nanoscale lipid compartmentalization. Whitebox in panels (**A**,**C**,**E**) shows the region that is magnified in panels (**B**,**D**,**F**).

Given their consistent morphology and spatial confinement within former adipocyte interiors, their biological significance warrants further investigation, potentially involving correlative immunolabeling or lipid-specific AFM modes. Figure 6 focuses on nanodomain structures (~200 nm), which may represent lipid compartmentalization or artifacts of defatting.

## 4. Relevance to Nephrotic Syndrome

Adipose tissue is highly dynamic in NS: the hypercatabolic state of NS induces large changes in body composition [4]. This hypercatabolic state is expected to impinge upon adipose tissue structure. Indeed, in chronic kidney disease, a smaller adipocyte size is found [3]. Furthermore, chronic kidney disease is accompanied by increased CD68-positive cells between adipocytes [3], which is intriguing due to the positivity for CD-68 of foamy cells in the kidney.

Unfortunately, data in NS are scanty. It is unclear if the hyperlipidemia characterizing NS increases lipid flux into adipocytes or is directly due to an increased release of lipids from adipocytes [60]. Similarly, while current technologies allow a striking characterization of fat microstructure and capillary vascularization, data in NS remain insufficient. Only in a few studies has capillary density been reported, such as a peritubular capillary rarefaction in congenital Finnish-type NS [61].

The occurrence of adipose tissue fibrosis in NS is also suggested by the increase in pro-fibrogenic cytokines (IL-1β and TGF-β1) in animal models of NS [62].

These structural features are often accompanied by extracellular matrix remodeling, which may contribute to increased tissue stiffness. Furthermore, endothelial dysfunction has been repeatedly demonstrated in chronic kidney disease [63], and this may manifest as reduced capillary density in adipose tissue.

Despite these known or inferred changes, systematic studies evaluating the multiscale structure of adipose tissue in NS remain limited. Most data are derived from animal models or inferred from systemic markers. Therefore, applying high-resolution imaging methods such as ultrasound, histology, and atomic force microscopy to adipose tissue in NS may reveal novel morphometric biomarkers reflective of disease burden or therapeutic response.

Our imaging framework could provide

- Quantitative metrics of adipocyte hypertrophy, cell packing, and vascular density;
- Markers of tissue stiffness or fibrosis inferred from surface wrinkling and nanostructures;
- Microvascular topology that reflects systemic endothelial health.

These data could help identify early or subclinical structural changes in adipose tissue during NS progression or treatment.

## 5. Discussion and Conclusions

This study presents a comprehensive multiscale imaging atlas of human adipose tissue from mesoscopic ultrasound to nanoscale atomic force microscopy, integrating innovative histological preparation techniques, aimed at improving our knowledge of the role of adipose tissue in NS.

High-frequency ultrasound successfully resolved the heterogeneity of adipose tissue, revealing speckle patterns that correspond to individual adipocytes. The application of computational filters, such as Voronoi tessellation, enhanced the interpretation of these echogenic borders, enabling non-invasive assessment of cellular organization in situ. This finding emphasizes ultrasound’s potential for functional tissue characterization beyond standard anatomical imaging.

The combination of rapid freezing and sequential solvent clearing preserved structural integrity while enhancing optical clarity. Importantly, polarized light microscopy revealed differential birefringence in capillaries, particularly in those devoid of red blood cells, allowing detailed mapping of the adipose microcirculation. The panoramic image stitching further contextualized microvascular patterns at scales rarely accessible in standard histology.

At the ultrastructural level, AFM imaging distinguished three topographical features: flat grainy surfaces within adipocyte interiors, small membrane wrinkles likely attributable to defatting-induced artifacts, and large, elongated wrinkles corresponding to inter-adipocyte capillaries. The latter showed periodic constrictions consistent with vascular wall stricture, suggesting potential biomechanical heterogeneity. These observations were validated through direct correlation with optical microscopy, confirming AFM as a powerful tool to investigate microvascular morphology in situ.

Together, these findings highlight a powerful, multiscale imaging strategy to investigate adipose tissue architecture in nephrotic syndrome from the cellular to the nanoscale level.

Finally, emerging electronic biosensing technologies offer new opportunities to complement imaging-based characterization of adipose remodeling in nephrotic syndrome. In particular, n-type organic field-effect transistors (OFETs) have recently been engineered for biosensing in aqueous environments, where they enable label-free detection of proteins, lipids, and cytokines with high sensitivity through modulation of charge transport at the semiconductor–electrolyte interface [64,65]. Because adipose tissue in nephrotic syndrome undergoes metabolic and inflammatory activation, leading to altered secretion of adipokines and circulating lipoproteins, OFET biosensors hold promise for real-time monitoring of these molecular signatures.

Future studies should focus on longitudinal imaging of adipose tissue in NS patients—before and after treatment such as corticosteroids or RAAS blockade—to correlate structural parameters with metabolic and renal outcomes.

## Figures and Tables

**Table 1:** Comparison of the lipidic asset between nephrotic syndrome and obesity-linked dyslipidemia. BMI: body mass index.

Characteristic	Nephrotic Syndrome	Obesity
Lipidic profile	Increased LDL, VLDL, triglycerides	Increased LDL, VLDL, triglycerides
Mechanism of dyslipidemia	Increased hepatic synthesis of albumin (secondary to proteinuria) and lipoproteins	Insulin resistance and consequent increased adipocyte lipolysis
Lipiduria	Present, typically HDL	Urinary Decanoilcarnitine is positively associated with BMI [42]; urinary steroid lipids increase in metabolic syndrome [43]
Mechanism of lipiduria	Possibly secondary to primitive glomerular damage	Unknown
Foamy cells (lipidic degeneration)	Typically, in kidney tubular cells	In atherosclerotic plaques
Kidney damage	Proteinuria is usually linked to increased risk of chronic kidney disease	Obesity is linked to increased risk of chronic kidney disease

**Table 2:** Available tools for the study of adipose tissue in vivo.

Method	Parameters
DXA; bioimpedentiometry; CT; MRI; US B-mode	Estimate of total and regional fat mass
US shear-wave elastometry	Fat “hardness” (fibrosis, dyslipidemia) [56]
Metabolomics of local adipose tissue	Extensive metabolic characterization of adipose tissue; requires an invasive approach [57] including tissue harvesting or local microdialysis [58]
Histology with standard microscopy; SEM and TEM	Gold standard for microscopic and ultrastructural characterization of fat tissue
**EMERGING METHODS**	
AFM	Allows for ultrastructural characterization of fat tissue without the limitations of TEM/SEM
Speckle characterization of adipose tissue in US	Promising method to gather information about fat structure [59]
AI analysis of histological slides with WSI	Allows for detection of features often missed by human eye

DXA: Dual-energy X-ray absorptiometry; CT: computed tomography; MRI: magnetic resonance Imaging; US: ultrasound; WSI: whole-slide imaging; SEM: scanning electron microscopy; TEM: transmission electron microscopy.

## Data Availability

No new data were created or analyzed in this study. Data sharing is not applicable to this article.
